# Mitochondrial DNA Depletion in Respiratory Chain–Deficient Parkinson Disease Neurons

**DOI:** 10.1002/ana.24571

**Published:** 2016-01-28

**Authors:** Anne Grünewald, Karolina A. Rygiel, Philippa D. Hepplewhite, Christopher M. Morris, Martin Picard, Doug M. Turnbull

**Affiliations:** ^1^ Wellcome Trust Centre for Mitochondrial Research and Medical Research Council/Biotechnology and Biological Sciences Research Council Centre for Ageing and VitalityInstitute of Neurosciences, Newcastle UniversityNewcastle upon TyneUnited Kingdom; ^2^ Institute of NeurogeneticsUniversity of LübeckLübeckGermany; ^3^ Molecular and Functional Neurobiology Group, Luxembourg Center for Systems BiomedicineUniversity of Luxembourg, Campus BelvalBelvauxLuxembourg; ^4^ Newcastle Brain Tissue Resource, Campus for Ageing and VitalityNewcastle UniversityNewcastle upon TyneUnited Kingdom; ^5^ Division of Behavioral Medicine, Department of Psychiatry and Department of Neurology, College of Physicians and SurgeonsColumbia University, Columbia University Medical CenterNew YorkNY

## Abstract

**Objective:**

To determine the extent of respiratory chain abnormalities and investigate the contribution of mtDNA to the loss of respiratory chain complexes (CI–IV) in the substantia nigra (SN) of idiopathic Parkinson disease (IPD) patients at the single‐neuron level.

**Methods:**

Multiple‐label immunofluorescence was applied to postmortem sections of 10 IPD patients and 10 controls to quantify the abundance of CI–IV subunits (NDUFB8 or NDUFA13, SDHA, UQCRC2, and COXI) and mitochondrial transcription factors (TFAM and TFB2M) relative to mitochondrial mass (porin and GRP75) in dopaminergic neurons. To assess the involvement of mtDNA in respiratory chain deficiency in IPD, SN neurons, isolated with laser‐capture microdissection, were assayed for mtDNA deletions, copy number, and presence of transcription/replication‐associated 7S DNA employing a triplex real‐time polymerase chain reaction (PCR) assay.

**Results:**

Whereas mitochondrial mass was unchanged in single SN neurons from IPD patients, we observed a significant reduction in the abundances of CI and II subunits. At the single‐cell level, CI and II deficiencies were correlated in patients. The CI deficiency concomitantly occurred with low abundances of the mtDNA transcription factors TFAM and TFB2M, which also initiate transcription‐primed mtDNA replication. Consistent with this, real‐time PCR analysis revealed fewer transcription/replication‐associated mtDNA molecules and an overall reduction in mtDNA copy number in patients. This effect was more pronounced in single IPD neurons with severe CI deficiency.

**Interpretation:**

Respiratory chain dysfunction in IPD neurons not only involves CI, but also extends to CII. These deficiencies are possibly a consequence of the interplay between nDNA and mtDNA‐encoded factors mechanistically connected via TFAM. ANN NEUROL 2016;79:366–378

Parkinson disease (PD) is a progressive movement disorder characterized by tremor, rigidity, bradykinesia, and postural instability that affects about 1% of those aged > 65 years.^1^ The pathological hallmarks of PD are selective loss of dopaminergic neurons and the presence of Lewy bodies in the substantia nigra (SN).^2^

Mitochondrial dysfunction has emerged as a potential mechanism in PD. Soon after the detrimental effects of 1‐methyl‐4‐phenyl‐1,2,3,6‐tetrahydropyridine on motor function were described and the inhibitory action of the toxin against respiratory chain complex I (CI) was unraveled,^3^ isolated CI deficiency was discovered in homogenates from postmortem SN samples of PD patients.^4^ The importance of this finding was emphasized when familial PD cases were found to harbor mutations in proteins involved in the removal of damaged mitochondria or the scavenging of reactive oxygen species that are predominantly generated by the electron transport chain.^5^

A study aiming to elucidate the molecular underpinnings of mitochondrial dysfunction in idiopathic PD (IPD) patients at the single‐cell level identified an accumulation of respiratory chain complex IV (CIV)‐deficient SN neurons with large somatic mtDNA deletions.^6^ Interestingly, the primary risk factor for PD development is ageing, which is itself correlated with the presence of CIV‐deficient neurons at similar levels to patients with IPD,^6^, ^7^ suggesting that age‐related damage accumulation could contribute to neuronal demise in IPD.^6^, ^7^

However, in the investigated IPD patients,^6^ CIV‐negative SN neurons constituted <3% of the total number of analyzed cells, a fraction well below the detection limit in traditional homogenate analysis. In comparison, the postmortem results from Schapira and colleagues^4^ imply an impairment of CI that is of sufficient magnitude to be detected in SN homogenates. Due to the lack of a robust histochemical method for the assessment of CI activity in single neurons, it has remained elusive whether mtDNA damage also underlies CI deficiency in IPD.

In this study, we aimed to determine the relative incidence of respiratory chain abnormalities and the molecular mechanisms underlying the loss of respiratory chain complexes in individual dopaminergic neurons from IPD patients. To establish the extent and relationship of these deficiencies in single neurons rather than SN homogenate, we employed quantitative quadruple immunofluorescence as an indicator of respiratory chain complex function at single‐cell resolution.^8^ In combination with laser‐capture microdissection (LCM) and real‐time polymerase chain reaction (PCR) analysis, this approach revealed altered levels of factors responsible for crosstalk between mitochondrial and nuclear compartments as a cause of respiratory chain dysfunction in IPD.

## Subjects and Methods

### Human Brain Tissue

Brain tissue was obtained from the Newcastle Brain Tissue Resource with ethical approval. Sections from paraffin‐embedded midbrain tissue of 10 IPD patients and 10 age‐matched controls (patients, mean ± standard error [SE]: 75.0 ± 1.8 years; controls, mean ± SE: 76.1 ± 4.1 years; *p* = 0.80) with comparable postmortem interval (PMI; patients [5 of them female], mean ± SE: 38.7 ± 4.5 hours; controls [4 of them female], mean ± SE: 45.8 ± 7.5 hours; *p* = 0.43) were used in this study. IPD patients had a mean (±SE) age at disease onset of 65.9 ± 1.2 years. Clinical and neuropathology information is summarized in the Table. Sections were cut at 5 μm using a microtome (Microm International, Walldorf, Germany) and mounted onto SuperFrostTM slides (Thermo Fisher Scientific, Waltham, MA).

**Table 1:** Clinical and Neuropathology Features of IPD Patients

Patient	Sex	Age at Onset, yr	Age at Death, yr	Clinical IPD	LB Pathology	Neuron Loss	Additional Features
IPD1	M	65	67	Affected	Positive	+++	Mild cognitive impairment, depression, anxiety
IPD2	M	66	77	Affected	Positive	+++	Dementia, visual hallucinations
IPD3	M	71	79	Affected	Positive	+++	Dementia, strokelike episodes, episodes of delirium
IPD4	M	66	81	Affected	Positive	+++	Cognitive impairment, visual hallucinations, confusion
IPD5	M	69	76	Affected	Positive	+++	Mild cognitive impairment, visual hallucinations, confusion
IPD6	F	60	65	Affected	Positive	++	Mild cognitive impairment, depression, visual hallucinations, anxiety
IPD7	F	62	77	Affected	Positive	++	Depression, anxiety, agitation
IPD8	F	62	71	Affected	Positive	+++	Mild cognitive impairment, psychosis, complex hallucinations (visual, auditory, olfactory, and tactile)
IPD9	F	66	81	Affected	Positive	+++	Dementia, impaired consciousness, visual and auditory hallucinations
IPD10	F	72	76	Affected	positive	++	Confusion, dyskinesia
Average ± SE		65.9 ± 1.2	75.0 ± 1.8				

++ = moderate; + ++ = severe; F = female; IPD = idiopathic Parkinson disease; LB = Lewy body; M = male; SE = standard error.

For mtDNA analysis in single cells, sections were cut from frozen midbrain blocks on a cryostat (Bright Instruments, Huntingdon, UK) at a thickness of 15 μm. Sections were stored at −80 °C, thawed, and allowed to air dry for 60 minutes at room temperature (RT) before immunohistochemistry was performed.

### Immunofluorescence Detection of Target Proteins in Formalin‐Fixed Brain Slices

We have previously established quantitative quadruple‐immunofluorescence as a sensitive tool to assess respiratory chain deficiencies at the single‐neuron level in postmortem tissue.^8^ In this study, this approach was applied to paraffin‐embedded midbrain sections using the following primary antibodies (all Abcam, Cambridge, MA) to target different subunits of respiratory chain complexes: CI, anti‐NDUFB8 (NADH dehydrogenase 1 beta subcomplex 8; ab110242) and anti‐NDUFA13 (NADH dehydrogenase 1 alpha subcomplex 13; ab110240); CII, anti‐SDHA (succinate dehydrogenase complex subunit A; ab14715); CIII, anti‐UQCRC2 (ubiquinol–cytochrome c reductase core protein II; ab14745); and CIV, anti‐COXI (cytochrome c oxidase subunit I; ab14705). The selected subunits are situated in functional domains correlating with the overall abundance of the respiratory chain complexes.^8^, ^9^, ^10^, ^11^, ^12^, ^13^ Additional mitochondrial antibodies included: anti‐porin (Abcam, ab14734), anti‐GRP75 (heat shock 70kDa protein 9 [mortalin]; Abcam, ab53098), anti‐TFAM (mitochondrial transcription factor A; Abcam, ab47517), and anti‐TFB2M (mitochondrial transcription factor B2; Sigma, St Louis, MO; HPA030265). To identify dopaminergic neurons, a rabbit anti–TH antibody (tyrosine hydroxylase; Sigma, T8700) was employed. Imaging using Axio Imager M1 (Zeiss, Oberkochen, Germany), and subsequent densitometric analysis of the acquired images was conducted as detailed in Grünewald et al.^8^ To rule out that the samples' PMIs negatively impacted on the abundance of the target proteins, we performed Spearman correlation analyses with PMI delay and mitochondrial protein levels as variables. This did not show a significant decline in protein levels as a result of higher PMI intervals.

### Immunofluorescence Detection of Target Proteins in Frozen Brain Slices

In formalin‐fixed sections, the yield and quality of mtDNA are reduced to such an extent that molecular analyses, especially in single cells, are impeded. To simultaneously determine the cellular abundance of CI and the levels of mtDNA in single SN neurons from IPD patients and controls, we optimized our immunofluorescence protocol for use with frozen brain tissue. The previously employed anti‐porin and anti‐GRP75 antibodies showed nonspecific labeling in frozen midbrain sections and therefore could not be used. Furthermore, anti‐NDUFA13, which provided a stronger signal in frozen tissue, was preferred over anti‐NDUFB8 in this experiment. In view of the results from fixed tissue, we considered the mitochondrial mass unchanged in IPD patients and carried out immunofluorescence labeling to detect NDUFA13 only. Sections were dried for 60 minutes at RT, fixed in 4% (wt/vol) paraformaldehyde for 10 minutes, then washed in H_2_O for 10 minutes and in Tris‐buffered saline containing 3% Tween‐20 (TBST) for 5 minutes. Sections were blocked in TBST and 1% normal goat serum (NGS) for 1 hour at RT. Anti‐NDUFA13 was diluted at 1:100 in blocking solution, and slides were incubated overnight at 4 °C. Following the primary antibody incubation, sections were washed 3 times in TBST for 5 minutes. An anti‐mouse IgG2b secondary antibody (A‐21141; Life Technologies, Carlsbad, CA) was diluted at 1:100 in 1% NGS TBST and applied for 60 minutes at RT. Following 3 washes in TBST, sections were incubated in Sudan black solution for 10 minutes to quench autofluorescence. Finally, sections were washed in H_2_O for 10 minutes to remove Sudan black and air dried.

### Mapping of SN Neurons for Isolation from Brain Slices by LCM

Following densitometric analysis of all neuromelanin‐positive SN neurons, neurons within a midbrain section were classified according to their absolute NDUFA13 levels. Ten neurons with high and 10 neurons with significantly (*p* < 0.0004) lower NDUFA13 levels but comparable neuromelanin content (as determined by densitometric analysis of the monochrome bright‐field image) were identified. These neurons were then mapped in a high‐resolution bright‐field image of the entire nigral region for each subject. The neurons of interest were located in the brain section, laser‐microdissected using a PALM MicroBeam (Zeiss), captured individually in 10 μl of lysis buffer, and incubated as previously described.^14^ This procedure was performed for 5 randomly selected IPD patients (compare in the Table: IPD2, IPD6, IPD7, IPD9, and IPD10).

### Mitochondrial DNA Deletion, Copy Number, and Replication Analysis

A triplex real‐time PCR assay for simultaneous detection of 3 targets within the mitochondrial genome (*ND1* [NADH dehydrogenase 1], *ND4*, and D‐loop) was performed.^15^ The quantification of *ND1* and *ND4* was conducted as previously published.^16^ For mtDNA copy number quantification, the *ND1* concentration per unit area was calculated. To detect mtDNA major arc deletions, the *ND1:ND4* was determined. The third target amplified in the triplex assay was located within an area of the noncoding region that is preserved in 99% of deleted mtDNA species reported to date.^15^, ^17^, ^18^ During transcription and replication, a linear DNA molecule, that is, 7S DNA, is incorporated in this particular region of the mitochondrial genome, resulting in a triple‐stranded structure denominated as “D‐loop.”^17^, ^19^ The D‐loop:*ND1* ratio is therefore representative of the mtDNA replication status in a given cell, with a higher ratio representing more actively transcribed or replicating mtDNA molecules.^20^ Two microliters of cell lysates from individual SN neurons were used per real‐time PCR reaction, and all measurements were performed in triplicates. Standard curves were generated by serial dilution of a DNA plasmid containing a single copy of each mtDNA target.^15^

To explore a link between the mitochondrial genome and CI abundance at the single‐cell level, the mtDNA deletion levels, copy numbers, and replication efficiency were quantified in individually laser‐captured NDUFA13‐positive and NDUFA13‐negative patient SN neurons.

Moreover, to obtain a general picture of mtDNA integrity and copy number in patients and controls, pooled SN neurons from 10 controls and 9 IPD patients (frozen midbrain sections were unavailable for IPD5) were analyzed using the same real‐time PCR assay. Thirty randomly selected SN neurons containing comparable neuromelanin levels were isolated from each tissue section as described and captured in a single tube in 20 μl lysis buffer. The experiment was conducted on 3 separate occasions per subject.

### Statistical Analysis

Densitometric analyses of individual SN neurons resulted in non‐normally distributed data in some cases. To account for this distribution, median values per person were calculated. In each run, the same control served as internal standard and its median abundance or median ratio of abundances was set to 100%. The differences between average patient and control group values in respect to protein abundances or mtDNA deletion levels, copy number, and mtDNA transcription/replication efficiency were assessed using unpaired 2‐sample *t* tests. To compare the *ND1*:area and D‐loop:*ND1* ratios in individually laser‐captured CI‐deficient or CI‐normal neurons isolated from the same individual, paired 2‐sample *t* tests were employed. Finally, the Spearman rank correlation coefficient (*r* ^2^), associated *p*‐value, and frequency distributions were calculated to evaluate the relationship between different parameters in the study. For statistical analysis, Prism 6.0 software (GraphPad, San Diego, CA) was used.

## Results

### CI and CII Deficiency in Single Nigral Neurons from IPD Patients

**Figure 1:**
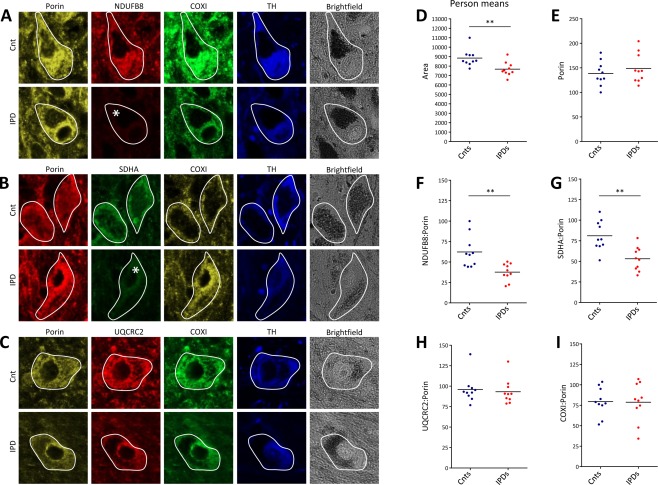
Abundances of subunits of respiratory chain complexes CI–IV and mitochondrial mass in dopaminergic substantia nigra (SN) neurons from idiopathic Parkinson disease (IPD) patients and controls (Cnt). (A–C) Epifluorescence microscopy after quadruple‐label immunofluorescence indicated reduced levels of the CI subunit NDUFB8 and the CII subunit SDHA in IPD patient neurons, whereas the CIII subunit UQCRC2, the CIV subunit COXI, and the mitochondrial mass marker porin were unchanged. The investigated neurons were tyrosine hydroxylase (TH) ‐positive and contained neuromelanin deposits indicative of their dopaminergic character. Deficiencies are marked by an asterisk. (D) Despite their significantly smaller size, (E) densitometric analysis of area‐normalized porin levels indicated unchanged mitochondrial mass in patient neurons. In agreement with the results from the visual inspection, the average (F) NDUFB8:porin and (G) SDHA:porin ratios in IPD patient neurons were significantly reduced. By contrast, (H) UQCRC2:porin and (I) COXI:porin ratios were comparable in control and patient SN neurons. Lines indicate mean group ratios. **p < 0.01. COXI = cytochrome c oxidase subunit I; NDUFB8 = NADH dehydrogenase 1 beta subcomplex 8; SDHA = succinate dehydrogenase complex subunit A; UQCRC2 = ubiquinol–cytochrome c reductase core protein II.

IPD patient characteristics are presented in the Table. Cell size analysis, immunofluorescence detection, and densitometric quantification of mitochondrial protein levels were performed in individual SN neurons from 10 IPD patients and 10 age‐matched controls (Fig 1). For each investigated parameter, means per person were derived. Calculation of the mean cell area showed that patient neurons were significantly smaller than control neurons. By contrast, the assessment of the mitochondrial mass marker porin at the single‐neuron level indicated comparable mitochondrial densities in patients and controls.

Next, we quantified the average neuronal protein abundance of the CI subunit NDUFB8 relative to mitochondrial mass (NDUFB8:porin ratio) in the patients and controls. In agreement with previous immunohistochemical studies,^9^, ^21^ we detected a significant reduction (60.5% of the control level) of NDUFB8 levels in the patient neurons (see Fig 1). Subsequently, we determined the abundance of the CII subunit SDHA. CII is the only complex of the electron transport chain entirely composed of nuclear‐encoded subunits. Quantification of SDHA:porin ratios in the SN neurons of all individuals showed a decrease in the amount of CII (65.5% of the control level) in patients. By contrast, assessment of the UQCRC2:porin ratio in individual neurons indicated no difference in the average CIII levels between patients and controls. Similarly, comparable CIV levels were found in neurons from patients and controls as quantified by the COXI:porin ratio.

### Relationship of CI, CII, and CIV in Single IPD Patient Neurons

**Figure 2:**
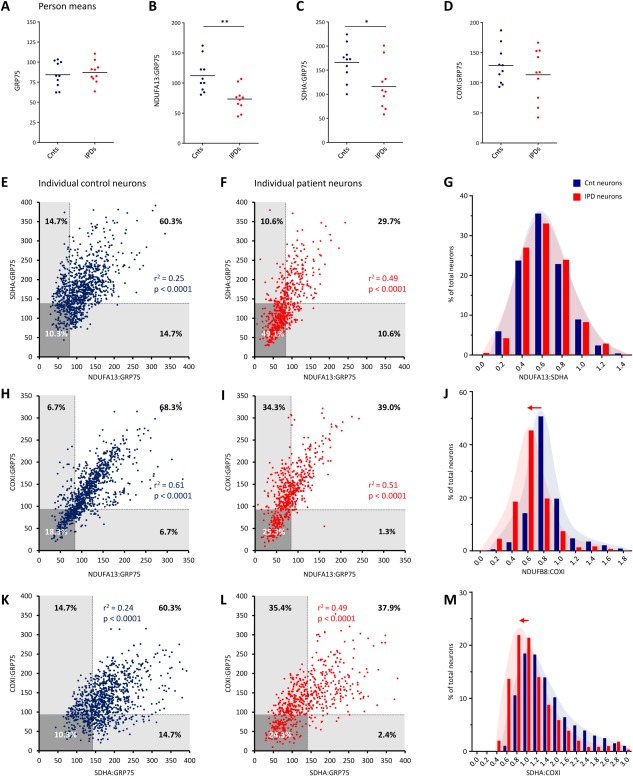
Dependencies between respiratory chain complexes CI, CII, and CIV in single control (Cnt) and idiopathic Parkinson disease (IPD) patient substantia nigra (SN) neurons. (A–D) Densitometric analysis of the mitochondrial mass marker GRP75, the CI subunit NDUFA13, the CII subunit SDHA, and the CIV subunit COXI in patient and control SN neurons. Calculation of mean neuronal protein abundances per person confirmed reduced levels of mitochondria‐normalized CI and CII in IPD patients. (E) Spearman correlation analysis of NDUFA13:GRP75 and SDHA:GRP75 ratios indicated a weak link between the protein levels of both complexes in individual control neurons. The lower 25th percentile of the control ranges for NDUFA13:GRP75 and SDHA:GRP75 were used to define thresholds of deficiency (*broken lines* and *shaded areas*). (F) In patient neurons, the association between NDUFA13:GRP75 and SDHA:GRP75 was increased. Applying the deficiency thresholds, 49.1% of cells lacked both NDUFA13 and SDHA. Equal proportions of cells (10.6%) showed either isolated NDUFA13 or SDHA deficiency. (G) This caused overlapping frequency distributions of NDUFA13:SDHA in patient and control cells. (H, I) Spearman correlation analysis of NDUFA13:GRP75 and COXI:GRP75 revealed coregulation of CI and CIV in individual control and patient neurons. About a quarter of patient cells (25.3%) were deficient for NDUFA13 and COXI. In 34.3% of all patient neurons, isolated NDUFA13 deficiency occurred, whereas isolated COXI deficiency was only present in 1.3% of cells. (J) An overall decrease in NDUFA13 resulted in a shift of the NDUFA13:COXI ratio in IPD patient neurons relative to the control population. (K) Spearman correlation analysis of the SDHA:GRP75 and COXI:GRP75 ratios indicated a weak dependency between CII and CIV in control cells. (L) In patient neurons, this coregulation increased as a result of the combined loss of CII and CIV in 24.3% of cells. A large proportion of patient SN neurons (35.4%) also showed isolated SDHA deficiency, (M) causing the frequency distribution of SDHA:COXI to shift toward lower SDHA levels. *p < 0.05, **p < 0.01. COXI = cytochrome c oxidase subunit I; GRP75 = heat shock 70kDa protein 9 (mortalin); NDUFA13 = NADH dehydrogenase 1 alpha subcomplex 13; *r* ^2^ = Spearman correlation coefficient; SDHA = succinate dehydrogenase complex subunit A. [Color figure can be viewed in the online issue, which is available at www.annalsofneurology.org.]

To correlate CI, CII, and CIV abundancies in single IPD patient and control neurons, we immunolabeled the CI subunit NDUFA13 along with SDHA (CII) and COXI (CIV). To simultaneously assess mitochondrial mass in neurons in this experimental setting, the anti‐porin antibody was replaced by an antibody against the outer mitochondrial membrane protein GRP75.^22^ Thus, antibody isotype mismatches between anti‐NDUFB8 and anti‐SDHA as well as between anti‐porin and anti‐NDUFA13 were circumvented. Results obtained using the anti‐GRP75 antibody confirmed that mitochondrial content is similar in patients and controls (Fig 2). Quantification of the mean ratios per person of NDUFA13:GRP75, SDHA:GRP75, and COXI:GRP75 also confirmed reduced neuronal CI and CII abundances as well as normal CIV levels in patients.

Regarding the relationship of CI and CII, Spearman correlation analysis revealed that the variance of NDUFA13:GRP75 accounts for 25% of the variance of SDHA:GRP75 in single control neurons (*r* ^2^ = 0.25, *p* < 0.0001; see Fig 2). In patient neurons, this interdependency was increased to 49% (*r* ^2^ = 0.49, *p* < 0.0001). To explore the link between loss of CI and CII in IPD in more detail, we defined objective thresholds for mitochondria‐normalized NDUFA13 and SDHA. SN neurons that fell below the 25th percentile of the control range for NDUFA13:GRP75 or SDHA:GRP75 were considered to express low levels of the respective respiratory chain subunit and are denominated as “deficient” hereafter. Interestingly, 49.1% of all IPD patient SN neurons lacked both CI and CII. Patient cells lacking only CI (10.6%) or only CII (10.6%) were equally frequent, resulting in overlapping frequency distributions of NDUFA13:SDHA for patient and control neurons.

**Figure 3:**
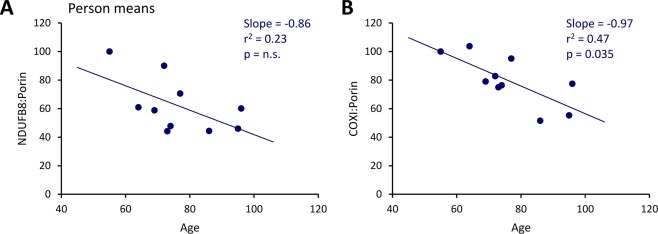
Correlation of respiratory chain complex abundances in substantia nigra neurons of controls with age. Plotting of the average neuronal NDUFB8:porin (A) and COXI:porin (B) against age at death revealed age‐associated declines. COXI = cytochrome c oxidase subunit I; NDUFB8 = NADH dehydrogenase 1 beta subcomplex 8; n.s. = not significant; *r* ^2^ = Spearman correlation coefficient. [Color figure can be viewed in the online issue, which is available at www.annalsofneurology.org.]

Because CIV deficiency has previously been implicated in IPD, we also wanted to explore the relationships between CIV and CI, as well as between CIV and CII at the single‐neuron level. In respect to CI and CIV, Spearman correlation analysis showed that 61% of the variance of NDUFA13:GRP75 was explained by the variance of COXI:GRP75 in single control neurons (*r* ^2^ = 0.61, *p* < 0.0001; see Fig 2). In patient neurons, this correlation was slightly reduced to 51% (*r* ^2^ = 0.51, *p* < 0.0001). Employing the above‐described strategy to establish deficiency cutoffs for NDUFA13:GRP75 and COXI:GRP75, 34.3% of patient neurons showed NDUFA13 deficiency without COXI involvement. About a quarter of the cells (25.3%) lacked both NDUFA13 and COXI. By contrast, neurons with isolated COXI deficiency were extremely rare (1.3%). In agreement with these observations, an overall shift of the NDUFB8:COXI ratio toward COXI was detected in IPD patient SN neurons. Further supporting a coregulation of CI and CIV, the average neuronal abundance of mitochondrial‐normalized NDUFB8 and COXI showed a similar rate of decline with age in controls (Fig 3).

Assessment of the relationship between CII and CIV (ie, SDHA:GRP75 and COXI:GRP75) indicated little correlation in control neurons (*r* ^2^ = 0.24, *p* < 0.0001; see Fig 2) but a markedly increased dependency in patient neurons (*r* ^2^ = 0.49, *p* < 0.0001), suggesting enhanced coregulation of CII and CIV in IPD patient neurons. The majority of deficient patient neurons lacked either SDHA and COXI (24.3%) or SDHA alone (35.4%). By contrast, isolated COXI deficiency was rare (2.4%), resulting in a shift of the SDHA:COXI ratio toward COXI.

### CI Deficiency Coincides with Loss of TFAM and TFB2M

**Figure 4:**
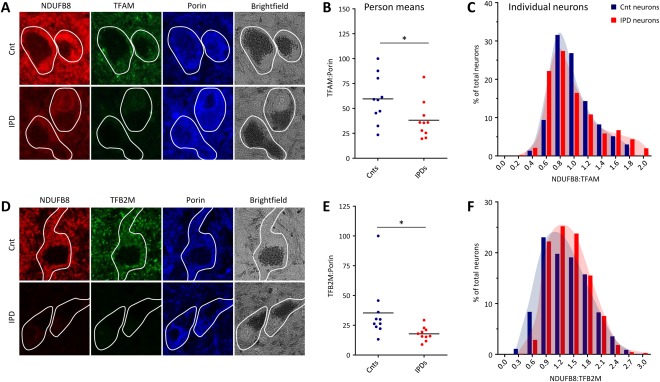
Abundance of mtDNA‐associated TFAM and TFB2M in patient and control (Cnt) substantia nigra neurons. (A) Epifluorescence microscopy after triple‐label immunofluorescence indicated joint loss of NDUFB8 and TFAM in idiopathic Parkinson disease (IPD) patients, whereas porin staining showed normal mitochondrial mass. (B) Densitometric analysis of investigated proteins confirmed significantly reduced TFAM:porin ratios in IPD patients. Lines represent mean group ratios. (C) In patients and controls, NDUFB8 and TFAM strongly correlated at the single‐neuron level, resulting in similar behaviors of the frequency histograms for NDUFB8:TFAM. (D) Triple‐label immunohistochemistry showed reduced abundances of NDUFB8 and TFB2M but normal porin levels in IPD neurons. (E) Densitometric analysis of average neuronal TFB2M and porin levels in patients and controls revealed a significant reduction of TFB2M:porin in the former. (F) The frequency distribution of the NDUFB8:TFB2M ratio in single neurons showed a slight shift toward lower TFB2M in patient cells. *p < 0.05. NDUFB8 = NADH dehydrogenase 1 beta subcomplex 8; TFAM = mitochondrial transcription factor A; TFB2M = mitochondrial transcription factor B2.

Next, we explored whether the loss of the partially mtDNA‐encoded CI is associated with impaired mtDNA transcription in IPD. The expression of mtDNA‐encoded subunits of the respiratory chain is controlled by TFAM.^23^, ^24^ TFAM protein abundance was quantified along with NDUFB8 and porin in the same cells. The average neuronal TFAM:porin ratio per person indicated significantly (*p* = 0.02) reduced protein levels of the transcription factor in IPD patients (64.0% of the control level) compared to controls (Fig 4A, B). In addition, the NDUFB8:porin and TFAM:porin ratios in single neurons were strongly correlated for both patients (*r* ^2^ = 0.74, *p* < 0.0001) and controls (*r* ^2^ = 0.72, *p* < 0.0001). The relative frequency of neurons with different NDUFB8:TFAM ratios was comparable in patients and controls, as indicated by overlapping frequency distributions (see Fig 4C).

To further ascertain a possible link between mtDNA transcription and CI deficiency in IPD, the protein levels of another mitochondrial transcription factor, TFB2M,^25^ were assessed. As for TFAM, the average neuronal TFB2M:porin ratio per person was significantly (*p* = 0.04) reduced in IPD patients (50.3% of the control level) compared to controls (see Fig 4D, E). Furthermore, mitochondria‐normalized NDUFB8 and TFB2M were moderately and equally correlated in individual IPD patient (*r* ^2^ = 0.57, *p* < 0.0001) and control (*r* ^2^ = 0.54, *p* < 0.0001) SN neurons, resulting in similar frequency distributions of NDUFB8:TFB2M (see Fig 4F).

### mtDNA Depletion in IPD Patient Neurons

In addition to their role as transcription factors, TFAM and TFB2M are involved in mitochondrial genome packaging^26^, ^27^ and replication.^23^, ^28^ Consequently, we sought to test whether loss of either factor is associated with mtDNA copy number and stability in SN neurons of IPD patients.

**Figure 5:**
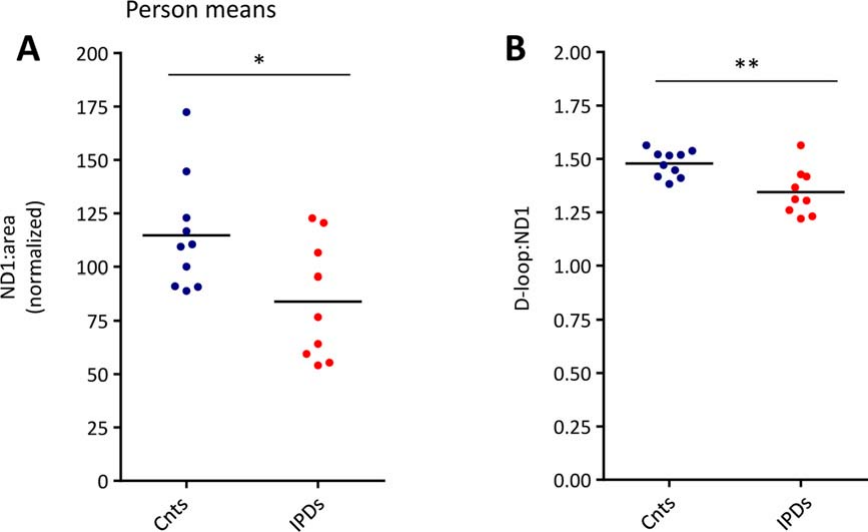
mtDNA analysis in pooled substantia nigra neurons isolated from control (Cnt) and idiopathic Parkinson disease (IPD) patient midbrain sections. Simultaneous real‐time polymerase chain reaction quantification of mtDNA fragments in *ND1* and the D‐loop in 30 neurons per person and experiment from 10 controls and 9 IPD patients are shown. This analysis indicated significantly reduced (A) mtDNA copy numbers (*ND1*:area) and (B) D‐loop:*ND1* ratios in IPD patients compared to controls. *p < 0.05, **p < 0.01. *ND1* = NADH dehydrogenase 1. [Color figure can be viewed in the online issue, which is available at www.annalsofneurology.org.]

Real‐time PCR quantification of the mitochondrial copy number (*ND1*:area) in pooled SN neurons from controls and patients showed significantly (*p* = 0.026) fewer mtDNA copies in IPD patient cells (73.1% of the control level; Fig 5A). In addition, using our triplex real‐time PCR assay, we measured the levels of 7S DNA, which associates with the mitochondrial genome in the D‐loop region in a transcription/replication‐dependent manner. The D‐loop:*ND1* ratio indicated significantly (*p* = 0.004) fewer 7S DNA molecules per mitochondrial genome in patient neurons (90.9% of the control level; see Fig 5B), linking mtDNA depletion to impaired mtDNA replication in IPD. Of note, the prevalence of mtDNA deletions in pooled neurons did not differ between patients and controls (data not shown).

### MtDNA Depletion Correlates with CI Deficiency

**Figure 6:**
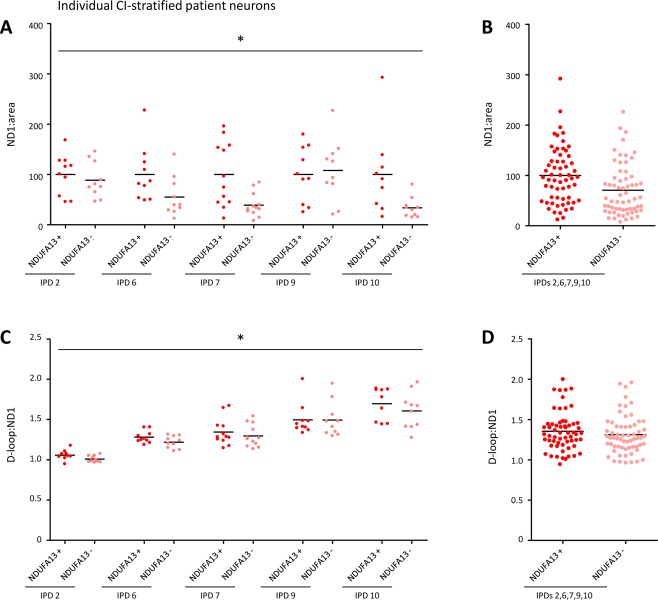
mtDNA real‐time polymerase chain reaction analysis in individually isolated patient substantia nigra neurons with high or low CI abundance. (A) Determination of the *ND1*:area ratio in single NDUFA13‐positive and NDUFA13‐negative neurons from 5 idiopathic Parkinson disease (IPD) patients showed significantly reduced mtDNA copy numbers in the latter cells. (B) On average, NDUFA13‐negative patient neurons contained 29.4% fewer mtDNA copies than NDUFA13‐positive patient neurons. (C, D) Impaired mtDNA transcription/replication was indicated by a significant reduction in the D‐loop:*ND1* ratio in NDUFA13‐negative neurons of all patients. *p < 0.05. *ND1* = NADH dehydrogenase 1; NDUFA13 = NADH dehydrogenase 1 alpha subcomplex 13. [Color figure can be viewed in the online issue, which is available at www.annalsofneurology.org.]

To explore whether the observed mtDNA depletion in IPD patient SN neurons correlates with CI deficiency at the single‐cell level, we isolated individual NDUFA13‐positive and NDUFA13‐negative neurons from 5 IPD patients. Real‐time PCR analysis revealed significantly (*p* = 0.041) lower mtDNA copy numbers (*ND1*:area) in NDUFA13‐deficient neurons (Fig 6). On average, NDUFA13‐negative patient neurons harbored 29.4% fewer mtDNA copies per cell area than NDUFA13‐positive patient neurons. Consistent with this result, quantification of mtDNA‐associated 7S DNA indicated significantly (*p* = 0.014) fewer mtDNA transcription/replication events in NDUFA13‐negative IPD patient neurons. By contrast, mtDNA major arc deletions did not correlate with CI abundance in individually laser‐captured patient neurons (data not shown).

## Discussion

Applying quadruple immunofluorescence to postmortem SN sections, we performed a detailed single‐neuron level characterization of respiratory chain deficiency in IPD. This high‐resolution quantitative approach enabled us to evaluate the relationship between the mitochondria‐normalized abundances of respiratory chain CI–IV. First, we confirmed experimental results from the late 1980s indicating CI (nicotinamide adenine dinucleotide ubiquinone oxidoreductase) deficiency in SN homogenates in IPD.^4^ In our hands, this deficiency in individual IPD SN neurons corresponded to a loss of CI (NDUFB8 and NDUFA13) protein levels normalized for mitochondrial mass. We furthered our analysis by quantifying the abundance of a subunit of CII (SDHA), which represents the only respiratory chain complex entirely composed of nuclear‐encoded proteins. To our surprise, IPD patient SN neurons contained significantly lower levels of SDHA, suggesting an involvement of both nuclear and mitochondrial factors in IPD pathogenesis. By contrast, the average protein levels of subunits of respiratory chain CIII (UQCRC2) and CIV (COXI) were comparable in nigral neurons of IPD patients and aged controls. CI and CII deficiencies occurred in the absence of changes in neuronal mitochondrial content, thus implicating specific molecular mechanisms that result in qualitative mitochondrial respiratory chain deficiency.

In addition to the well‐established CI deficit, also CIV dysfunction has previously been implicated in the pathogenesis of IPD.^6^, ^7^ Benefiting from the high specificity and resolution of our single‐cell immunofluorescence approach, we studied the quantitative relationships between CI, CII, and CIV as an index of their coregulation in SN neurons. In agreement with the common dependency of CI and CIV assembly and function on the mitochondrial genome,^29^ NDUFA13 and COXI abundances were strongly correlated in single control and patient neurons. The majority of dysfunctional patient SN neurons demonstrated either isolated CI, or combined CI and CIV deficiencies, with few cells exhibiting selective loss of CIV. From this observation, we deduced that CIV deficiency in nigral neurons only occurs in the presence of CI deficiency in IPD. This molecular signature may arise from mtDNA alterations as discussed below.

CI and CII as well as CIV and CII were loosely linked in control neurons. However, in patient SN neurons, the associations between NDUFA13 and SDHA as well as between COXI and SDHA increased to the level of coregulation of NDUFA13 and COXI. The joint occurrence of CI, CII, and CIV deficiencies in IPD patient neurons (despite none of the subunits of CII being encoded by the mitochondrial genome) suggested that nuclear signaling pathways, such as the nuclear respiratory factor (NRF)1/2 pathway, are involved in the loss of respiratory chain complexes. The NRF1/2 pathway^30^, ^31^ not only controls the transcription of all CII subunits (and of some subunits of CI and CIV) but also directly regulates the mRNA expression of the mtDNA transcription factors TFAM^24^ and TFB2M.^28^

One reason why CII deficiency was not detected in a previous study on postmortem midbrain sections from the Newcastle PD cohort may be different methodology.^6^ Whereas our new image analysis method allows detection of subtle changes in protein levels within each tested neuron in a quantitative manner, previously applied visual classification schemes distinguished only between enzymatically completely SDH‐negative and ‐positive neurons. The same holds true for COXI deficiency in single neurons. Employing quantitative immunofluorescence, we detected subtle CIV deficits in SN neurons of aged controls that appear to even out the previously observed small shift toward CIV deficiency in the PD patients.^6^

To test the potential role of nuclear signaling in IPD further, we investigated the abundance of TFAM and TFB2M in nigral neurons from patients and controls. Both proteins correlated with CI levels at the single‐cell level and were reduced in patient tissue, implicating downregulated transcription of mtDNA genes as a potential factor in the development of CI deficiency in IPD. However, both TFAM and TFB2M are also required for transcription‐primed replication of the mitochondrial genome,^24^, ^28^ opening the possibility that mtDNA depletion contributes to respiratory chain deficiency in IPD.

To explore this hypothesis, we performed real‐time PCR analysis of the mitochondrial genome in pooled patient and control SN neurons. Patient neurons contained fewer mtDNA copies and less mtDNA replication/transcription‐associated 7S DNA than control cells. Further linking mtDNA depletion to loss of CI in IPD, individually isolated CI‐deficient patient neurons contained even fewer mtDNA copies and 7S molecules than their counterparts with normal CI levels. In keeping with these findings, increased exposure of the mitochondrial genome to TFAM has been reported to enhance 7S DNA association and mtDNA replication in an in organello DNA synthesis system.^32^ In line with our hypothesis stating that TFAM deficiency induces mtDNA depletion in IPD, conditional silencing of *TFAM* provokes PD‐like features such as respiratory chain dysfunction, degeneration of dopaminergic SN neurons, and progressive impairment of motor function in mice.^33^, ^34^ Remarkably, in addition to its effect as a CI inhibitor, the PD‐inducing reagent 1 methyl‐4‐phenylpyridinium also interferes with mtDNA replication by destabilization of the mitochondrial D‐loop.^35^, ^36^ Hence, our molecular results converge with these findings to indicate that toxin‐induced parkinsonism and the actual movement disorder may share a common molecular origin, namely disrupted mtDNA transcription and replication.

It is curious why CI and CII are seemingly more affected by impaired nuclear transcription activation and mtDNA depletion in IPD SN neurons than CIII and CIV. This discrepancy, however, was previously observed in a histopathology study on Alper syndrome, a primary mtDNA replication defect. In these individuals with a mutation in the mtDNA polymerase gamma (*POLG*) gene, mtDNA depletion resulted in isolated CI (but not CIV) deficiency.^37^ Preferential degradation of CI in Alper syndrome and of CI and CII in IPD may be due to lower total abundances of these complexes in respect to CIII and CIV. The stoichiometric ratios of the respiratory chain complexes have been measured as CI, 0.8–1.3; CII, 1.2–1.4; CIII, 3; and CIV, 6.0–7.5 in bovine heart mitochondria.^38^ Thus, CIV may exist in excess, whereas CI levels may be limiting. Moreover, the quantification of respiratory chain protein turnover rates in mice showed that the subunits of CII have the shortest average half‐life, followed by the components of CI and CIV, whereas the components of CIII have the longest half‐life.^39^ The high stability of the CIII subunits potentially explains why this complex behaves differently from CI and CII, despite relatively similar stoichiometric proportions. An alternative scenario underlying our immunohistochemical and real‐time PCR results may be that SN neurons lacking functional CIII and CIV (and harboring deletions) are more prone to degeneration and are thus undetectable in postmortem tissue. This would lead to a selection bias where CIII and CIV deficiencies would escape our analysis, yielding the current histopathological picture where CI and CII deficiencies predominate.

Whereas our study exclusively focused on respiratory chain deficiencies in dopaminergic neurons of the SN, it would be interesting to explore whether the described respiratory chain and mtDNA phenotypes extend to other areas of the brain known to be affected in the early and late phases of IPD. According to the staging scheme by Braak and colleagues,^40^ neuronal pathology in IPD evolves from the olfactory bulb and the dorsal motor nucleus via the locus ceruleus and the SN to the basal forebrain and amygdala, finally reaching the medial temporal lobe structures and the cerebral cortex.^40^, ^41^ Single‐cell analyses in these specific brain regions would help to further elucidate the contribution of mitochondrial dysfunction to disease progression.

In summary, our findings link respiratory chain deficiencies to both nDNA and mtDNA elements in nigral neurons of IPD patients. Possibly as a consequence of impaired NRF1/2 signaling, the transcription of nuclear‐encoded subunits of the respiratory chain complexes and of the mitochondrial transcription factors TFAM and TFB2M is compromised in IPD. Lack of TFAM packaging of the mitochondrial genome causes D‐loop destabilization and hampers mtDNA transcription and replication. In turn, a reduction in mtDNA copy number and transcripts potentiates respiratory chain dysfunction, which is primarily reflected in the loss of CI. By contrast, CIV deficiency appears only together with CI deficiency, possibly representing a later phase of respiratory chain impairment that may precipitate neuronal loss. Although mtDNA deletions have previously been associated with CIV deficiency in IPD, our results reveal that quantitative differences in mtDNA abundance and replication more specifically discriminate between CI‐normal and CI‐deficient SN neurons. Our detailed single‐cell analysis of respiratory chain proteins further expands the mitochondrial phenotype of IPD to include CII deficiency, which largely correlates with the loss of CI. The heretofore‐suspected predominant defect of CI is likely only the most pronounced sign of a manifold picture of respiratory chain complex deficiencies arising from impaired mitochondrial–nuclear signaling in IPD.

## Author Contributions

Concept and study design: A.G., K.A.R., D.M.T. Data acquisition and analysis: A.G., P.D.H., M.P., C.M.M. Drafting the manuscript and figures: A.G., K.A.R., M.P., D.M.T.

## Potential Conflicts of Interest

Nothing to report.
